# Ribosome Biogenesis Underpins Tumor Progression: A Comprehensive Signature for Survival and Immunotherapy Response Prediction

**DOI:** 10.3390/cancers17152576

**Published:** 2025-08-05

**Authors:** Amr R. Elhamamsy, Salma M. Aly, Rajeev S. Samant, Lalita A. Shevde

**Affiliations:** 1 Department of Pathology, University of Alabama at Birmingham, Birmingham, AL 35294, USA; amrrafat@uab.edu (A.R.E.); rajeevsamant23@gmail.com (R.S.S.); 2 Department of Family and Community Medicine, University of Alabama at Birmingham, Birmingham, AL 35294, USA; smaly@uabmc.edu; 3 O’Neal Comprehensive Cancer Center, University of Alabama at Birmingham, Birmingham, AL 35294, USA

**Keywords:** ribosome biogenesis, prognostic biomarker, LASSO regression, immunotherapy, cancer stemness, tumor microenvironment, TCGA, METABRIC

## Abstract

**Background**: RiBi is integral to cell proliferation, and its dysregulation is increasingly recognized as a hallmark of aggressive cancers. We sought to develop and validate a composite “PanRibo-515 score” reflecting RiBi activity across multiple tumor types, assess its prognostic significance, and explore its relationship with immune checkpoint therapy outcomes. **Methods**: We curated 515 RiBi–associated genes (PanRibo-515) and used a LASSO regression-based strategy on a training dataset (GSE202203) to select the prognostically most relevant subset of 68 genes (OncoRibo-68). Directionality (positive or negative impact on survival) was assigned based on the sign of the LASSO coefficients. We integrated a forward selection approach to identify a refined subset of genes for computing the OncoRibo-68 score. For validation, patients in The Cancer Genome Atlas (TCGA) were stratified into high or low OncoRibo-68 score groups for survival analyses. Additional validation for immunotherapy response was conducted using bioinformatic platforms used for immunotherapy response analysis. **Results**: A higher OncoRibo-68 score consistently correlated with poorer overall and progression-free survival across multiple cancers. Elevated OncoRibo-68 score was linked to an immunosuppressive tumor microenvironment, but interestingly to increased response to checkpoint inhibitors. **Conclusions**: Our findings highlight RiBi as an important determinant of tumor aggressiveness and identify the OncoRibo-68 score as a promising biomarker for risk stratification and therapy selection. Future research may evaluate whether targeting RiBi pathways could enhance treatment efficacy, particularly in combination with immunotherapy.

## Simple Summary

Ribosome Biogenesis (RiBi) is the process by which cells create the molecular machinery needed to assemble amino acids into proteins. Because cancer cells divide quickly, they often have higher RiBi activity; this can drive their aggressiveness and reduce patients’ survival. To understand this better, we developed RiBi scores (PanRibo-515 and OncoRibo-68) that capture overall RiBi activity in tumors. We tested both scores in several cancer patient datasets, including those with immunotherapy treatments, and found that higher scores were linked to worse outcomes. By measuring key genes in RiBi, we can more accurately predict which tumors are likely to progress faster or respond to immunotherapy. This research could help with identification of patients who need alternate treatment approaches or could be matched to targeted therapies, ultimately improving patient care.

## 1. Introduction

Cancer progression is fundamentally driven by a complex interplay between oncogenic signaling, cellular metabolism, and the tumor microenvironment [1]. Among these processes, RiBi—the synthesis and assembly of ribosomal components—has emerged as a crucial yet often underappreciated contributor of tumor development [2,3]. Rapidly proliferating cancer cells frequently upregulate RiBi to meet their heightened demand for protein synthesis, and this increased activity can be linked to aggressive phenotypes, therapeutic resistance, and poor clinical outcomes [4]. Despite a growing interest in targeting RiBi therapeutically, the precise role and prognostic value of RiBi-related genes across different cancer types remains an active area of investigation [5,6].

Recent work has shed light on how altered ribosomal protein abundance and rRNA processing may serve as biomarkers and drivers of malignancy [7]. For instance, some studies suggest that disruptions in the nucleolar architecture can activate oncogenic pathways including MYC or interfere with tumor suppressors such as p53 [8,9]. However, the underlying mechanisms by which elevated RiBi informs worse survival or increased metastatic potential are still debated [1,10]. Conflicting findings from various experimental models highlight the need for a robust, integrative approach that captures the cumulative contribution of multiple RiBi-related genes rather than focusing on a single component [11].

Here, we propose a PanRibo-515 score derived from a curated set of 515 RiBi-associated genes that evaluate overall RiBi activity across multiple cancer types. By applying feature selection (LASSO) and carefully assigning directionality (i.e., whether higher expression of a gene predicts better or worse outcomes), we distilled this large gene set into a streamlined and prognostically relevant signature (OncoRibo-68). We evaluated the OncoRibo-68 score in major clinical datasets (including GSE202203, and TCGA), testing its association with diverse outcomes such as overall survival, progression-free interval, and immunotherapy response. Our findings suggest that heightened RiBi activity, as reflected by our composite score, correlates with tumor aggressiveness, immune evasion, and diminished survival. We further explore how integrating the OncoRibo-68 score with standard immuno-oncology biomarkers might refine patient stratification and guide treatment decisions. Thus, our study aims to establish a robust framework for estimating RiBi activity in solid tumors and to underscore its prognostic and potential therapeutic value.

## 2. Materials and Methods

### 2.1. Data Collection and Preprocessing

We identified 515 genes associated with RiBi via comprehensive literature and database searches (PubMed, Gene Ontology [12], GeneCards [13], UniProt [14], OMIM [15], and PANTHER [16]) and used them as our candidate RiBi gene set. We then retrieved normalized gene expression and clinical data from multiple sources:

- GSE202203 (training cohort): Expression data restricted to the 515 RiBi genes were acquired from the Gene Expression Omnibus (GEO).
- The Cancer Genome Atlas (TCGA): Processed gene expression and matched clinical outcomes for pan-cancer analyses were accessed via the University of California, Santa Cruz Xena Browser [17].

All datasets were checked for completeness, and missing expression or clinical values were omitted or set to zero only when essential for consistent downstream analysis. Genes or samples with insufficient data were excluded from the final analysis.

### 2.2. OncoRibo-68 Score Construction via LASSO Regression

To narrow down the 515-gene set, we applied LASSO (Least Absolute Shrinkage and Selection Operator) regression and forward selection strategies directly on the complete GSE202203 dataset to identify the most prognostically relevant subset of ribosome biogenesis genes, focusing on overall survival (OS) as the outcome variable. LASSO was implemented in Python (version 3.10; scikit-learn library version 1.X) using 5-fold cross-validation (LassoCV). Genes with non-zero coefficients were retained. The sign of each retained coefficient determined whether higher expression predicted poorer survival (“negative” predictor) or better survival (“positive” predictor). A forward selection approach was then applied to identify an optimal subset of 68 genes (OncoRibo-68), maximizing the coefficient of determination (R^2^).

For each selected gene, we assigned a score of +1 or −1 in each sample based on whether its expression exceeded (or did not exceed) the median expression level, with the sign of the contribution dependent on the directionality inferred from the LASSO coefficient. The OncoRibo-68 score for each sample was computed as the sum of these ±1 values across all selected genes. OncoRibo-68 score was subsequently validated using independent external cohort (TCGA BRCA).

### 2.3. Survival Analyses

Kaplan–Meier (KM) analyses were conducted to evaluate the prognostic impact of the RiBi scores (PanRibo-515 and OncoRibo-68 scores) on multiple survival endpoints (e.g., overall survival, disease-specific survival, disease-free interval, and progression-free interval). For most cohorts, we stratified patients into two groups (e.g., High vs. Low RiBi scores) by either:

- Median cut-off: Patients with scores above the median were assigned to the High group, and those below the median to the Low group.
- Quartile-based cut-offs: Patients in the top quartile vs. bottom quartile of the RiBi scores distribution.

Survival curves were generated in GraphPad Prism (version 10) or, where noted, in Python using the lifelines package (version 0.30.0). The log-rank test was used to compare survival distributions between groups, and hazard ratios (HR) with 95% confidence intervals were reported.

### 2.4. Statistical Analyses

All statistical analyses, unless otherwise specified, were performed in GraphPad Prism (version 10). Two-sided *p*-values less than 0.05 were considered significant. Correlations between RiBi scores and continuous variables, including immune infiltration scores, aneuploidy, and stemness indices, were assessed using Pearson correlation analyses performed with GraphPad Prism (version 10) and Python’s pandas (version 2.2.3) and numpy (version 2.0.0.) libraries. Normality of the continuous variables was verified by the Kolmogorov–Smirnov test prior to correlation analysis. For multiple correlation analyses, *p*-values were adjusted using the false discovery rate (FDR) correction. In cases involving multiple group comparisons (e.g., ANOVA-based analyses), significance was determined using Tukey’s post hoc test, with adjusted *p*-values similarly indicated.

### 2.5. Immunotherapy Response and TIDE Analysis

We assessed the predictive value of the OncoRibo-68 score against known immunotherapy biomarkers using the TIDE (Tumor Immune Dysfunction and Exclusion) web tool (http://tide.dfci.harvard.edu/) [18]. TIDE compares user-defined biomarkers—submitted as a single or weighted gene set—to standard immunotherapy biomarkers (e.g., CD8, IFNG, TIDE, Merck18 TIS, MSI score) by generating receiver operating characteristic (ROC) curves and area under the curve (AUC) values. TIDE also provides KM plots for various checkpoint inhibitor–treated cohorts (anti–PD-1, anti–PD-L1, and anti–CTLA-4), enabling direct comparisons of survival outcomes between high and low OncoRibo-68 score groups.

### 2.6. Kaplan–Meier Plotter (Immunotherapy Module)

For further validation in immunotherapy-treated cohorts (e.g., melanoma, urothelial cancer), we employed the KMplot immunotherapy module (https://kmplot.com/) [19]. We uploaded the genes of the OncoRibo-68 score and assigned them a calculated weight according to their LASSO directionality and generated survival curves stratifying patients into “High” vs. “Low” expression groups. HRs and log-rank *p*-values were automatically calculated by the platform, and patient subgroups were defined based on the median or quartile cut-off per the tool’s default or user-defined settings.

### 2.7. Immune and Stemness Analyses

Immune-cell composition was estimated with the immunedeconv R package (Version 2.0.3), which applies transcriptomic deconvolution algorithms to bulk RNA-seq data. For human tumors we used the QuanTIseq engine as the primary method and confirmed key findings with EPIC, MCP-counter, xCell, CIBERSORT, ABIS. In parallel, tumor-purity metrics (Stromal and ESTIMATE scores) [20], and RNA-based stemness indices (RNAss) were obtained from, or recalculated with, UCSC Xena pipelines. Pearson correlation was then used to quantify the association between the RiBi scores and each immune-cell fraction, purity metric, or stemness index, and the results were visualized as scatterplots with best-fit regression lines or as correlation heat-maps.

### 2.8. Software and Code Availability

All Python analyses, including the LASSO-based feature selection, forward selection, survival modeling (lifelines), and visualizations (matplotlib, seaborn), were performed in Python 3.10 on a Windows 10 platform. Survival curves and additional statistical comparisons were generated or refined in GraphPad Prism (version 10). Custom Python (version 3.10) scripts used to compute RiBi scores, perform survival analyses, and integrate multiple datasets are available upon request.

## 3. Results

### 3.1. Comprehensive Profiling of RiBi-Related Genes Reveals Broad Prognostic Value

**Figure 1:**
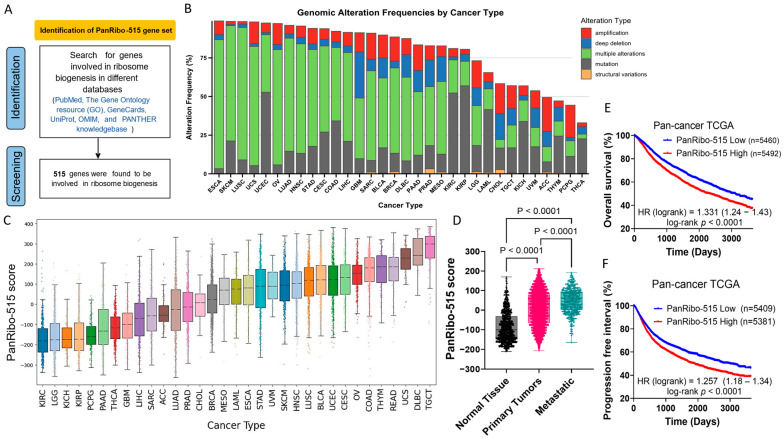
Development and prognostic implications of PanRibo-515 score. (**A**) Flowchart illustrates the approach for identifying PanRibo-515 gene set through comprehensive database screening (PubMed, Gene Ontology, GeneCards, UniProt, OMIM, and PANTHER). (**B**) Frequency of genetic and transcriptomic alterations in the PanRibo-515 gene set across multiple cancer types using cBioPortal, showing proportions of mutations (gray), copy number amplifications (red), deep deletions (blue), structural variations (orange) and multiple of these alterations (green). (**C**) Distribution of PanRibo-515 score in The Cancer Genome Atlas (TCGA) tumors, highlighting variability in RiBi activity across cancer types. (**D**) Comparison of PanRibo-515 scores among normal tissue, primary tumors, and metastatic lesions, showing a progressive increase with disease stage (*p* < 0.0001). (**E**) Kaplan–Meier curve for overall survival in pan-cancer TCGA cohorts, demonstrating worse prognosis for patients with high PanRibo-515 scores (HR = 1.369, log-rank *p* < 0.0001). (**F**) Kaplan–Meier curve for progression-free interval in the same cohort, revealing that a higher PanRibo-515 score is associated with significantly reduced progression-free times (HR = 1.274, log-rank *p* < 0.0001).

To investigate the significance of RiBi in cancer progression, we first identified 515 genes associated with RiBi through extensive database screening (Figure 1A). Across diverse tumor types, these genes exhibited frequent genetic and transcriptomic alterations, including copy number amplifications, deep deletions, and significant mRNA dysregulation (Figure 1B).

#### 3.1.1. PanRibo-515 Score Distribution and Prognostic Impact in TCGA

To quantify the collective impact of these 515 RiBi genes on tumor biology, we derived a PanRibo-515 score for each sample in The Cancer Genome Atlas (TCGA) by aggregating gene expression data. This score varied substantially across different cancer types (Figure 1C), underscoring the heterogeneous nature of RiBi dysregulation. Moreover, when comparing normal tissues, primary tumors, and metastatic lesions, there was an increase in the PanRibo-515 score with disease progression (Figure 1D; *p* < 0.0001), implying that heightened RiBi activity may be an important driver of tumor progression.

At the pan-cancer level, TCGA samples with higher PanRibo-515 scores had significantly reduced overall survival (HR = 1.369, log-rank *p* < 0.0001; Figure 1E) and shorter progression-free intervals (HR = 1.274, log-rank *p* < 0.0001; Figure 1F). These findings demonstrate that an upregulated RiBi program, assessed with the application of the PanRibo-515 score, is broadly linked to poor clinical outcomes, regardless of tumor type.

#### 3.1.2. Functional Enrichment of Genes in PanRibo-515 Signature

**Figure 2:**
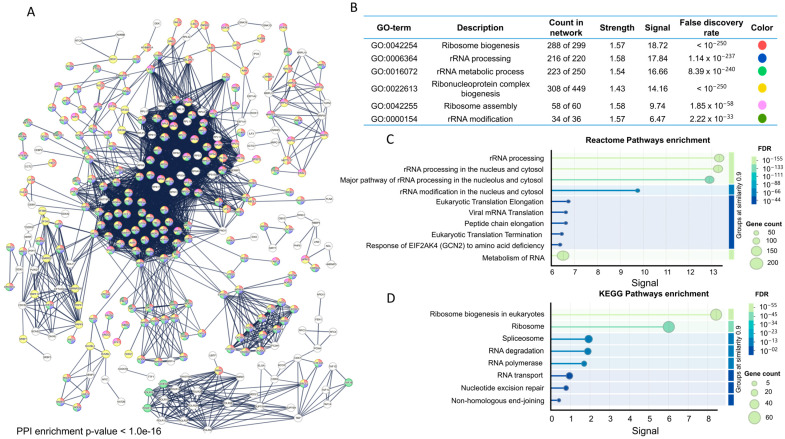
Protein–protein interaction network and functional enrichment analysis of PanRibo-515 gene set. (**A**) Comprehensive protein–protein interaction (PPI) network of PanRibo-515 gene set generated in STRING database (average node degree: 26.7, local clustering coefficient: 0.57, enrichment *p*-value: <1.0 × 10^−16^). Nodes represent individual protein, and edges denote experimentally validated or predicted interactions, forming dense clusters associated with ribosome biogenesis. Color code indicates which proteins in the network belong to each GO term. (**B**) Top Gene Ontology (GO) terms enriched among PanRibo-515 gene set, highlighting significant involvement in key processes such as ribosome biogenesis, rRNA processing, and ribonucleoprotein complex assembly (false discovery rate < 1 × 10^−30^, calculated by Benjamini–Hochberg correction). Color code indicates which proteins in the network belong to each GO term. (**C**) Reactome pathway enrichment analysis, revealing prominent pathways linked to rRNA processing, eukaryotic translation, and viral mRNA translation, underscoring the pivotal role of PanRibo-515 gene set in both canonical and stress-responsive translational pathways. (**D**) KEGG pathway enrichment analysis indicating substantial overrepresentation of PanRibo-515 gene set in pathways relevant to ribosome biogenesis, spliceosome function, and RNA metabolism. The size of the data points in (**C**,**D**) correspond to the number of genes mapped to each pathway, and the color gradient indicates the false discovery rate (FDR).

To gain deeper mechanistic insights, we constructed a protein–protein interaction (PPI) network with all 515 genes in PanRibo-515 gene set using STRING (Figure 2A). This revealed dense clusters of interacting proteins, indicating extensive functional interdependencies within RiBi pathways. Gene Ontology (GO) analysis confirmed significant enrichment for rRNA processing, ribonucleoprotein assembly, and other ribosome-related processes (Figure 2B), while Reactome and KEGG pathway analyses highlighted the essential role of these genes in eukaryotic translation, viral mRNA translation, RNA metabolism, and spliceosome (Figure 2C,D). Altogether, these data validate that the PanRibo-515 gene set reflects fundamental mechanisms driving protein synthesis and suggests that dysregulation of these processes could promote tumor progression.

#### 3.1.3. PanRibo-515 Score Stratifies Survival Endpoints Across Multiple Tumor Types

To further substantiate the prognostic utility of the PanRibo-515 score, we performed Kaplan–Meier analyses for multiple survival endpoints—overall survival, disease-specific survival, disease-free interval, and progression-free interval—across various TCGA tumor types (Supplementary Figures S1–S4). In most cancers assessed, a high PanRibo-515 score (red curves) was consistently associated with worse patient outcomes than a low PanRibo-515 score (blue curves). Supplementary Figure S1 shows the detrimental impact of elevated RiBi activity on overall survival, while Supplementary Figures S2–S4 illustrate similar patterns for disease-specific, disease-free, and progression-free survival, respectively, across different types of cancer. These results underscore that tumor cells exhibit an upregulation of RiBi contributing to rapid proliferation, and potential immune evasion—that culminate in an increased likelihood of disease progression and mortality.

To uncover how the PanRibo-515 score drives transcriptional programs to impact the tumor immune microenvironment, we systematically used diverse deconvolution algorithms. Across independent deconvolution methods (Supplementary Figures S5 and S6), the PanRibo-515 score consistently correlates with (i) diminished cytotoxic/effector cells—CD8^+^ T, NK, γδ-T and M1 macrophages—most sharply in KIRC, LUAD/LUSC and BRCA; (ii) enrichment of suppressive myeloid elements (M2 macrophages, neutrophils, MDSC-like and non-classical monocytes), especially in GI, lung and breast tumors; and (iii) increased fibroblast, endothelial and overall stromal signatures that fortify an immune-excluding micro-environment.

Taken together, these analyses suggest that the PanRibo-515 gene set represents a key biological process—ribosome biogenesis—and may serve as a valuable pan-cancer prognostic tool. The association between a higher PanRibo-515 score and poorer survival outcomes across various cancers supports the potential role of ribosome biogenesis in tumor progression, thereby justifying further evaluation and refinement of this signature for possible clinical applications.

### 3.2. Elevated PanRibo-515 Score Is Linked to Aggressive Phenotypes and Immune Modulation in LIHC, KIRC, and LUAD

**Figure 3:**
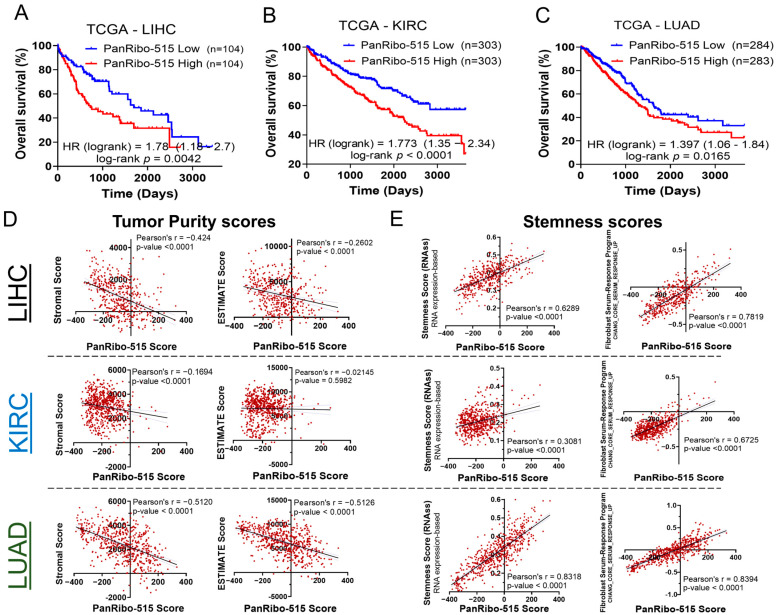
High PanRibo-515 score is associated with poorer outcomes and enhanced stemness in multiple tumor types. (**A**–**C**) Kaplan–Meier overall survival curves for patients with hepatocellular carcinoma (LIHC), kidney renal clear cell carcinoma (KIRC), and lung adenocarcinoma (LUAD) in the TCGA. Patients were stratified into “High” (red) and “Low” (blue) PanRibo-515 score groups by median PanRibo-515 value. Hazard ratios (HR) and log-rank *p*-values indicate a significantly worse prognosis for patients with a high PanRibo-515 score. (**D**) Scatter plots showing the relationship between PanRibo-515 score and tumor microenvironment purity (Stromal Score, ESTIMATE Score) in LIHC, KIRC, and LUAD. Pearson’s correlation coefficients (r) and *p*-values suggest that elevated PanRibo-515 scores correlate with reduced tumor purity. (**E**) Scatter plots illustrating a positive correlation between PanRibo-515 score and cancer stemness metrics (e.g., RNA expression–based stemness scores), indicating that tumors with higher RiBi activity also tend to exhibit more pronounced stemness characteristics.

Building on the pan-cancer results, we next focused on three tumor types—hepatocellular carcinoma (LIHC), kidney renal clear cell carcinoma (KIRC), and lung adenocarcinoma (LUAD)—to investigate how an elevated PanRibo-515 score influences patient outcomes and tumor biology. Across all three cancers, a high PanRibo-515 score (red curves) was significantly associated with worse overall and disease-specific survival, as illustrated by the Kaplan–Meier curves in Figure 3A–C and Supplementary Figure S7A (*p* < 0.05 for each comparison), collectively suggesting that tumors with RiBi hyperactivation exhibit clinical outcomes associated with aggressive behavior.

#### 3.2.1. Correlation of PanRibo-515 Score with Tumor Purity and Stemness

Further analyses revealed that an increased PanRibo-515 score was inversely correlated with tumor purity. Specifically, Stromal and ESTIMATE scores both tended to be higher when PanRibo-515 scores were lower, indicating that tumors with high RiBi activity may harbor a denser or more complex microenvironment. Representative scatter plots for LIHC, KIRC, and LUAD confirm these findings in Figure 3D, where elevated PanRibo-515 scores correlate negatively with these metrics (Pearson’s *r* ranging from –0.18 to –0.42; *p* < 0.0001).

Notably, high PanRibo-515 scores also track closely with increased cancer stemness. As shown in Figure 3E, multiple stemness indices—such as the RNA expression–based stemness score and fibroblast stemness program—were positively correlated with PanRibo-515 scores (Pearson’s *r* up to ~0.76; *p* < 0.0001). This suggests that heightened RiBi may foster an environment conducive to stem-like properties, potentially fueling tumor initiation, therapeutic resistance, and relapse.

#### 3.2.2. Associations with Aneuploidy, Proliferation, and the Immune Microenvironment

To gain additional insight into the interplay between RiBi activity and tumor pathophysiology, we examined several key parameters (Supplementary Figure S7B–D). First, higher PanRibo-515 scores correlated strongly with aneuploidy (Pearson’s *r* up to 0.73; *p* < 0.0001), implying that dysregulated RiBi may coincide with genomic instability in these malignancies. Likewise, the PanRibo-515 score showed a positive association with a proliferation module score (Pearson’s *r* up to 0.53; *p* < 0.0001), consistent with the essential role of ribosomes in supporting rapid tumor growth.

In contrast, an elevated PanRibo-515 score tended to correlate negatively with the T-cell module score (Pearson’s *r* down to −0.50; *p* < 0.0001), suggesting that hyperactive RiBi can be associated with lower T-cell infiltration or function within the tumor microenvironment. This trend was further corroborated by a heatmap of Pearson correlation coefficients showing that the PanRibo-515 score is associated with low abundance of CD8^+^ T and NK cells, yet each tumor type mobilizes suppression differently (Supplementary Figure S8A–E). LIHC shows only a mild T-reg uptick and little stromal change, KIRC combines modest effector loss with strong T-reg/M2 enrichment and pronounced fibro-endothelial expansion, while LUAD displays the steepest effector deficit alongside a dominant M2–neutrophil bias but minimal stroma, raising the possibility that RiBi-driven mechanisms could impede antitumor immune responses.

Overall, these results reinforce the notion that a high PanRibo-515 score confers multiple features of tumor aggressiveness, including enhanced proliferation, genomic instability, diminished immune infiltration, and elevated stemness. By dissecting these associations in LIHC, KIRC, and LUAD, we highlight how an upregulated RiBi may underlie intrinsic tumor properties and extrinsic immune-modulatory effects, thereby shaping clinical outcomes in diverse malignancies.

### 3.3. Development and Validation of the OncoRibo-68 Score in Breast Cancer

**Figure 4:**
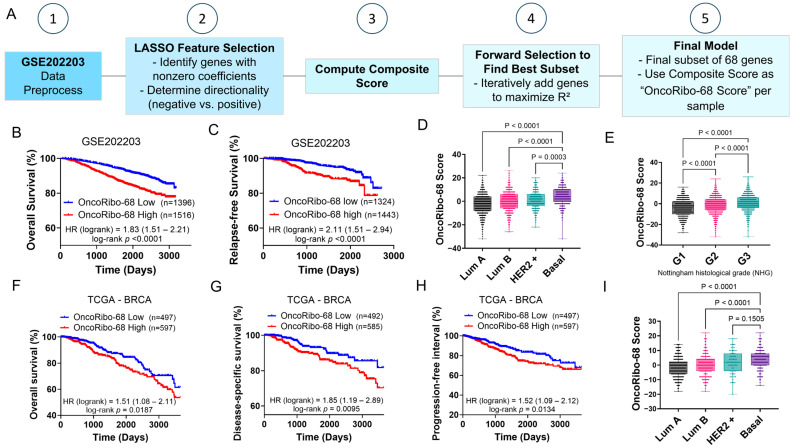
The Development and validation of OncoRibo-68 score in breast cancer cohorts. (**A**) Schematic of the stepwise approach used to develop OncoRibo-68 score from GSE202203 data: (1) data preprocessing, (2) LASSO feature selection and gene directionality determination, (3) computation of a composite ±1 score, (4) forward selection to maximize R^2^, and (5) derivation of OncoRibo-68 Score. (**B**,**C**) Kaplan–Meier analyses in the GSE202203 cohort, showing that higher OncoRibo-68 scores (red) are significantly associated with poorer overall survival (**B**) and relapse-free survival (**C**). Hazard ratios (HR) and log-rank *p*-values are indicated. (**D**,**E**) OncoRibo-68 score distribution by molecular subtype (**D**) and Nottingham histological grade (**E**) within GSE202203, demonstrating significant differences among subtypes (Luminal A, Luminal B, HER2+, and Basal) and among histological grades (G1, G2, G3). (**F**–**H**) Independent validation in TCGA breast cancer (BRCA) data: higher OncoRibo-68 scores (red) are associated with worse overall survival (**F**), disease-specific survival (**G**), and progression-free interval (**H**). (**I**) OncoRibo-68 score across major TCGA BRCA subtypes, showing again that basal breast cancers (the most aggressive subtype) have distinct score distributions compared to other subtypes. All survival curves were compared Via log-rank tests, and boxplots were analyzed using one-way ANOVA followed with Tukey’s multiple comparison test.

Having established the broad prognostic impact of RiBi across multiple solid tumors, we next focused on breast cancer to derive a more targeted gene set with enhanced clinical relevance. We used the GSE202203 cohort as a training dataset, employing a LASSO-based feature selection pipeline to narrow the original PanRibo-515 gene set to a smaller subset (Figure 4A). Specifically, we first identified genes with nonzero LASSO coefficients and determined the directionality of each gene’s association with survival (negative vs. positive). We then computed a composite ±1 score reflecting whether each gene’s expression exceeded or fell below its median expression, adjusting the sign based on each gene’s directionality. A forward selection approach was applied to iteratively optimize the model’s explanatory power (R^2^). This multistep process yielded the final OncoRibo-68 Score, whose development is detailed further in Supplementary Figure S9 (e.g., LASSO coefficient paths, residual plots, and calibration curves).

#### 3.3.1. Prognostic Significance of OncoRibo-68 Score in GSE202203

When applied to the GSE202203 cohort, OncoRibo-68 score demonstrated robust prognostic capacity. Patients with high OncoRibo-68 scores (red curves) experienced significantly worse overall survival (HR = 1.827, log-rank *p* < 0.0001; Figure 4B) and relapse-free survival (HR = 2.106, log-rank *p* < 0.0001; Figure 4C). Moreover, OncoRibo-68 score varied substantially by breast cancer subtype—with particularly high levels in the more aggressive HER2+ and Basal subtypes—and by Nottingham histological grade (G1 vs. G2 vs. G3), suggesting that upregulated RiBi might underlie more advanced tumor phenotypes (Figure 4D,E).

#### 3.3.2. Independent Validation in TCGA BRCA

We confirmed these findings in the TCGA breast cancer (BRCA) cohort, where again a high OncoRibo-68 score correlated with worse overall survival, disease-specific survival, and progression-free interval (Figure 4F–H). Notably, the Basal subtype displayed distinctly elevated OncoRibo-68 scores relative to Luminal A, Luminal B, and HER2+ subtypes (Figure 4I), reinforcing the notion that enhanced RiBi is a hallmark of particularly aggressive breast cancers.

#### 3.3.3. Functional Insights into the 68-Gene Signature

To elucidate the biological underpinnings of this refined signature, we generated a protein–protein interaction (PPI) network for the OncoRibo-68 gene set in STRING (Supplementary Figure S10A). The resultant network exhibited dense clustering, indicating a tightly interlinked set of RiBi functions. Furthermore, quartile-based survival analyses in both GSE202203 and TCGA BRCA confirmed the prognostic robustness of the OncoRibo-68 score (Supplementary Figure S10B–F), with patients in the top quartile of OncoRibo-68 expression faring significantly worse than those in the bottom quartile. These data highlight not only the importance of RiBi in breast tumor progression but also the practical utility of OncoRibo-68 score as an actionable prognostic and potentially predictive biomarker for breast cancer.

### 3.4. Predictive Potential of OncoRibo-68 Gene Set in Immunotherapy-Treated Patients

**Figure 5:**
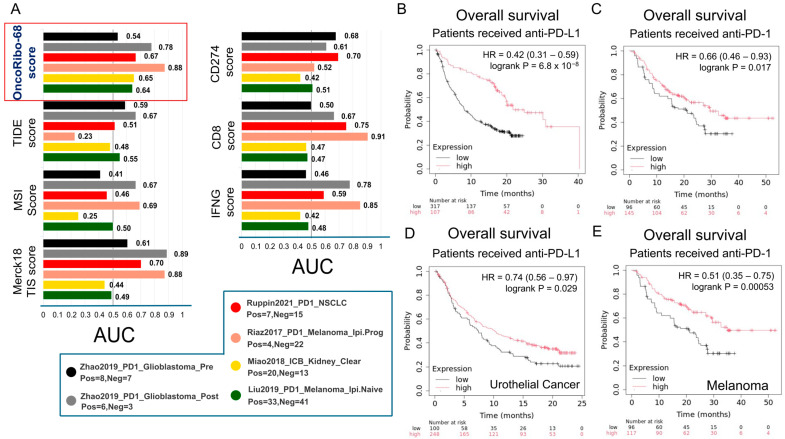
OncoRibo-68 score, and gene set compare favorably with established immunotherapy response signature and predict clinical outcomes in patients receiving immune checkpoint inhibitors. (**A**) Bar charts from the TIDE web tool (http://tide.dfci.harvard.edu/) showing predictive performance (AUC) for several known immunotherapy biomarkers (e.g., CD8, IFNG, MSI score, Merck18 TIS, TIDE) alongside OncoRibo-68 score (red). “Random” serves as a baseline reference. (**B**,**C**) Kaplan–Meier survival curves generated in KMplot (Immunotherapy module) for patients treated with anti–PD-L1 (**B**) or anti–PD-1 (**C**) therapy, stratified by high versus low OncoRibo-68 expression. Hazard ratios (HR) and log-rank *p*-values demonstrate improved overall survival in one of the OncoRibo-68 expression groups, indicating a potential predictive role of OncoRibo-68 score. (**D**,**E**) Additional KMplot analyses in urothelial cancer and melanoma cohorts, illustrating similarly significant survival differences based on the OncoRibo-68 genes expression level in patients receiving anti–PD-L1 or anti–PD-1 treatments.

To determine whether heightened RiBi also impacts responses to immune checkpoint inhibitors, we compared OncoRibo-68 score to established immunotherapy biomarkers/scores using the TIDE algorithm and KMplot immunotherapy modules. In Figure 5A, the OncoRibo-68 score demonstrated a robust predictive performance, achieving area under the ROC curve (AUC) values that were either comparable to or surpassed those of established immunotherapy biomarkers, including the CD8, IFNG, MSI, Merck18 TIS, and TIDE scores. These findings highlight the potential utility of the OncoRibo-68 score as a strong predictive marker for immunotherapy response across multiple patient cohorts.

#### 3.4.1. Clinical Outcomes in Patients Receiving Anti–PD-1 or Anti–PD-L1

In the KMplot analyses (Figure 5B,C), patients with high OncoRibo-68 gene expression experienced significantly better overall survival (OS) than those with low expression, suggesting that the RiBi signal can predict benefit—or lack thereof—from anti-PD-L1 or anti-PD-1 therapies. Similar patterns were observed in urothelial cancer and melanoma cohorts (Figure 5D,E), where Kaplan–Meier curves again revealed distinct survival trajectories based on RiBi expression levels. These findings imply that upregulated RiBi activity may shape the immunotherapy response, possibly by modulating the tumor microenvironment or influencing tumor–immune cell interactions.

#### 3.4.2. TIDE Analysis Across Multiple Immunotherapy Cohorts

To validate these observations across diverse datasets, we performed additional Kaplan–Meier analyses in Supplementary Figure S8, stratifying patients receiving immune checkpoint inhibitors (ICI) by OncoRibo-68 gene set using TIDE. Across the Liu2019, Gide2019, Miao2018, Mariathasan2018, and Zhao2019 studies, patients displaying elevated OncoRibo-68 genes expression often exhibited notably better survival outcomes, with Z-statistics and *p*-values reflecting the log-rank test comparisons (Supplementary Figure S11A–C). These collective results reinforce the notion that RiBi can meaningfully influence ICI efficacy.

In summary, these immunotherapy-focused analyses expand the clinical relevance of RiBi beyond prognostication alone. By comparing favorably with established immunotherapy biomarkers and showing strong correlations with OS in multiple ICI-treated cohorts, OncoRibo-68 signature emerges as a promising candidate for guiding patient selection and optimizing combination treatment strategies in the era of precision oncology.

## 4. Discussion

In this study, we undertook a comprehensive investigation into the role of RiBi in cancer, starting with a broad PanRibo-515 gene set and ultimately refining it into a robust 68-gene “OncoRibo-68 Score”. Our findings reveal several key insights. First, the dysregulation of RiBi appears to be a pervasive feature across multiple malignancies, as evidenced by PanRibo-515 score prognostic significance in lung, liver, and kidney cancers. Second, the RiBi score strongly correlates with tumor aggressiveness, immune cell infiltration, aneuploidy, and cancer stemness, suggesting that heightened RiBi both supports rapid tumor growth and shapes an immunosuppressive microenvironment. Finally, our development of the OncoRibo-68 score, specifically in the context of breast cancer, shows that a subset of RiBi genes can provide refined prognostic stratification and may even predict responsiveness to immunotherapies.

Ribosomes are central to protein synthesis, but recent studies have highlighted their broader involvement in tumorigenesis and metastasis [1,10]. Consistent with previous evidence, we found that increased expression of RiBi genes is linked to shorter overall survival and poorer progression-free outcomes in diverse cancers. This observation supports the hypothesis that hyperactive RiBi contributes not only to rapid tumor cell proliferation but also to broader oncogenic processes—such as metabolic reprogramming, stress adaptation, and evasion of immune surveillance [2,21]. Furthermore, an expanding body of work indicates that oncogenic drivers like MYC and mTOR can upregulate ribosomal RNA transcription, while tumor suppressors (e.g., p53) often act as “nucleolar guardians” to restrain ribosome production [7,9,22]. Our pan-cancer findings dovetail with these mechanisms, suggesting that tumors with high RiBi scores may preferentially leverage these pathways to gain a survival and proliferative advantage.

Our data show that an elevated RiBi score often inversely correlates with T-cell infiltration and tumor purity, while correlating positively with stemness signatures. These observations align with reports linking RiBi to immunomodulatory functions; for instance, high nucleolar activity can modulate the tumor stroma and reduce the effectiveness of cytotoxic T-cells [23,24,25,26]. Additionally, recent studies indicate that cancer stem cells may exploit increased ribosomal output to drive continuous self-renewal and sustain elevated protein synthesis demands during rapid replication [27,28,29]. Together, these findings suggest that targeting RiBi—perhaps by inhibiting upstream regulators such as RNA polymerase I—may not only blunt tumor growth but also reshape the tumor microenvironment to favor immune effector cells.

While the PanRibo-515 gene set functioned as a robust pan-cancer biomarker, focusing on breast cancer allowed us to refine this large signature into a smaller OncoRibo-68 gene set through LASSO-driven feature selection. Notably, high OncoRibo-68 scores consistently identified patients with poor survival metrics (overall, disease-specific, and relapse-free), highlighting this subset’s strong prognostic utility. This aligns with prior work in breast cancer, demonstrating that perturbations in ribosomal RNA modification correlate with tumor grade and subtype [30,31]. Interestingly, the most aggressive breast subtypes (e.g., basal-like) displayed the highest RiBi scores, underscoring the potential synergy between RiBi hyperactivity and molecular features that promote invasiveness and early relapse [32,33].

Our investigation into multiple immunotherapy cohorts (anti–PD-1 and anti–PD-L1 treatments) revealed that elevated RiBi expression levels or scores may associate with diminished therapeutic benefit, reflecting possible immune evasion or suppression. Other groups have similarly reported that an immunosuppressive tumor microenvironment can be sculpted, in part, by nucleolar stress responses and ribosome dysfunction [34,35,36]. Although the exact mechanisms require further elucidation, the superior predictive capability of the OncoRibo-68 score—demonstrated by its ability to outperform several established immunotherapy biomarkers, including CD8 T-cell, IFNG, MSI, TIDE, and Merck18 TIS scores—positions it as a highly promising candidate for further clinical validation and implementation. With immune checkpoint inhibitors at the forefront of cancer therapy, a more nuanced understanding of how RiBi intersects with immune regulation could guide patient selection or inform combination therapies that jointly target ribosomal pathways and immune checkpoints [37].

In the light of the promising nature of our findings, a few limitations warrant discussion. We performed extensive bioinformatic analyses on large public datasets; prospective validation in controlled clinical trials will be essential for translating the OncoRibo-68 score into a clinical tool. The mechanistic underpinnings linking RiBi to tumor stemness and immunomodulation remain incompletely understood. Additional in vitro and vivo studies could clarify whether specific gene clusters within our 68-gene signature directly modulate immune cell infiltration or metastatic potential. Finally, expansion of analyses beyond the cancers examined here will be an important consideration as certain tumor types can harbor distinct ribosomal assembly pathways or unique compensatory mechanisms.

## 5. Conclusions

In summary, our work underscores the pervasive role of RiBi in shaping cancer progression, emphasizing both proliferative and immunological dimensions. By integrating high-throughput transcriptomic datasets and applying stringent computational selection methods, we identified a refined OncoRibo-68 signature that offers robust prognostic power across multiple cohorts, most notably in breast cancer. Additionally, the observed correlations with immunotherapy outcomes suggest that targeting RiBi or incorporating OncoRibo-68 score into patient stratification may improve the effectiveness of current therapeutic regimens. Future studies to define the specific molecular interfaces between ribosomal pathways and immune checkpoints could accelerate the development of combination therapies and further precision medicine approaches in oncology.

## Data Availability

This research used public databases. Codes and scripts are available upon reasonable request.

## Supplementary Materials

The following supporting information can be downloaded at: https://www.mdpi.com/article/10.3390/cancers17152576/s1, Figure S1: Overall survival across variousTCGA tumor types stratified by a PanRibo-515 score; Figure S2: Disease-specific survival across TCGA tumor types stratified by PanRibo-515 score; Figure S3: Disease-free interval across TCGA tumor types stratified by PanRibo-515 score; Figure S4: Progression-free interval across TCGA tumor types stratified by PanRibo-515 score; Figure S5: Correlation of PanRibo-515 Score with Immune Cell Populations across Cancer Types; Figure S6: PanRibo-515 Score Associations with Immune Cell Subtypes across Cancer Types; Figure S7: PanRibo-515 score correlates with aneuploidy, proliferation, and immune cell profiles in LIHC, KIRC, and LUAD; Figure S8: Correlation of PanRibo-515 Score with Immune Cell Populations in LIHC, KIRC, and LUAD; Figure S9: LASSO-based model development and assessment using GSE202203 training data; Figure S10: Protein–protein interaction (PPI) network of OncoRibo-68 gene set and quartile-based survival analyses in GSE202203 and TCGA BRCA; Figure S11: Kaplan–Meier plots from TIDE analyses of multiple immunotherapy cohorts, stratified by the OncoRibo-68 gene set as a custom biomarker.

## Abbreviations

The following abbreviations are used in this manuscript:

FDR	False discovery rate
GEO	Gene Expression Omnibus
HER2	Human epidermal growth factor receptor 2
HR	Hazard ratio
ICI	Immune checkpoint inhibitor
KEGG	Kyoto Encyclopedia of Genes and Genomes
KIRC	Kidney renal clear cell carcinoma
KM	Kaplan–Meier
LASSO	Least absolute shrinkage and selection operator
LIHC	Liver hepatocellular carcinoma
LUAD	Lung adenocarcinoma
mTOR	mammalian target of rapamycin
MSI	Microsatellite instability
OMIM	Online Mendelian Inheritance in Man
OS	Overall survival
TP53	Tumor protein p53
PD-1	Programmed cell death protein 1
PD-L1	Programmed cell death-ligand 1
PPI	Protein–protein interaction
RiBi	RiBi
R^2^	Coefficient of determination
RNAss	RNA expression–based stemness
TCGA	The Cancer Genome Atlas
T-cell	T lymphocyte
TIDE	Tumor Immune Dysfunction and Exclusion
Xena	UCSC Xena browser
