# Supplementary material for: Ribosome Biogenesis Underpins Tumor Progression: A Comprehensive Signature for Survival and Immunotherapy Response Prediction
Source: Cancers (Basel). 2025 Aug 5;17(15):2576. doi: 10.3390/cancers17152576 (PMC12345896; doi:10.3390/cancers17152576)
Supplement: Supplementary file 1 [file cancers-17-02576-s001.zip › cancers-3772603-supplementary.pdf]

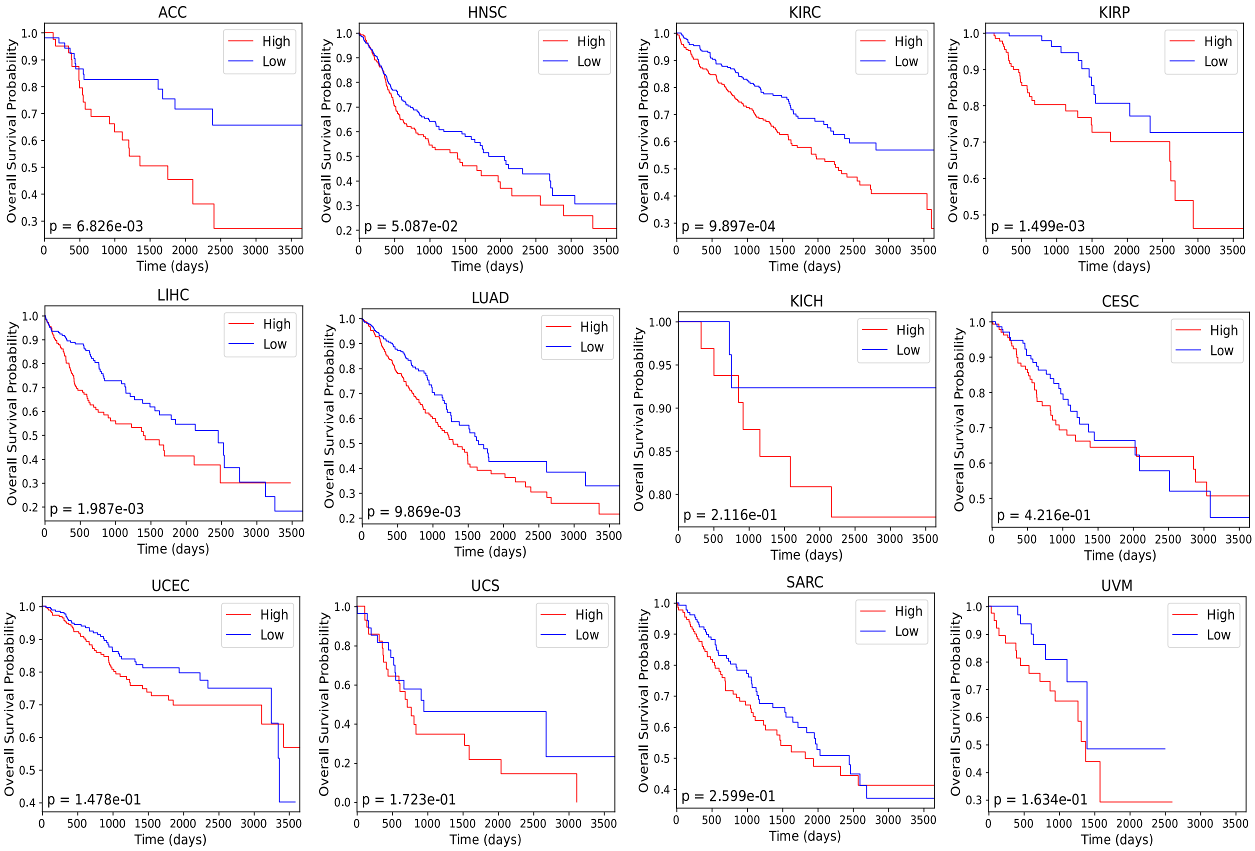

Supplementary Figure 1. Overall survival across various TCGA tumor types stratified by a PanRibo-515 score.

Each panel is titled with the respective tumor type. Patients were divided into “High” (red) and “Low” (blue) PanRibo-515 score groups based on the median PanRibo-515 score in each cancer. Kaplan–Meier curves were generated using Python’s lifelines package (alongside pandas, numpy, and matplotlib), and the p-values shown in each panel were calculated by log-rank tests to assess survival differences between the two groups.

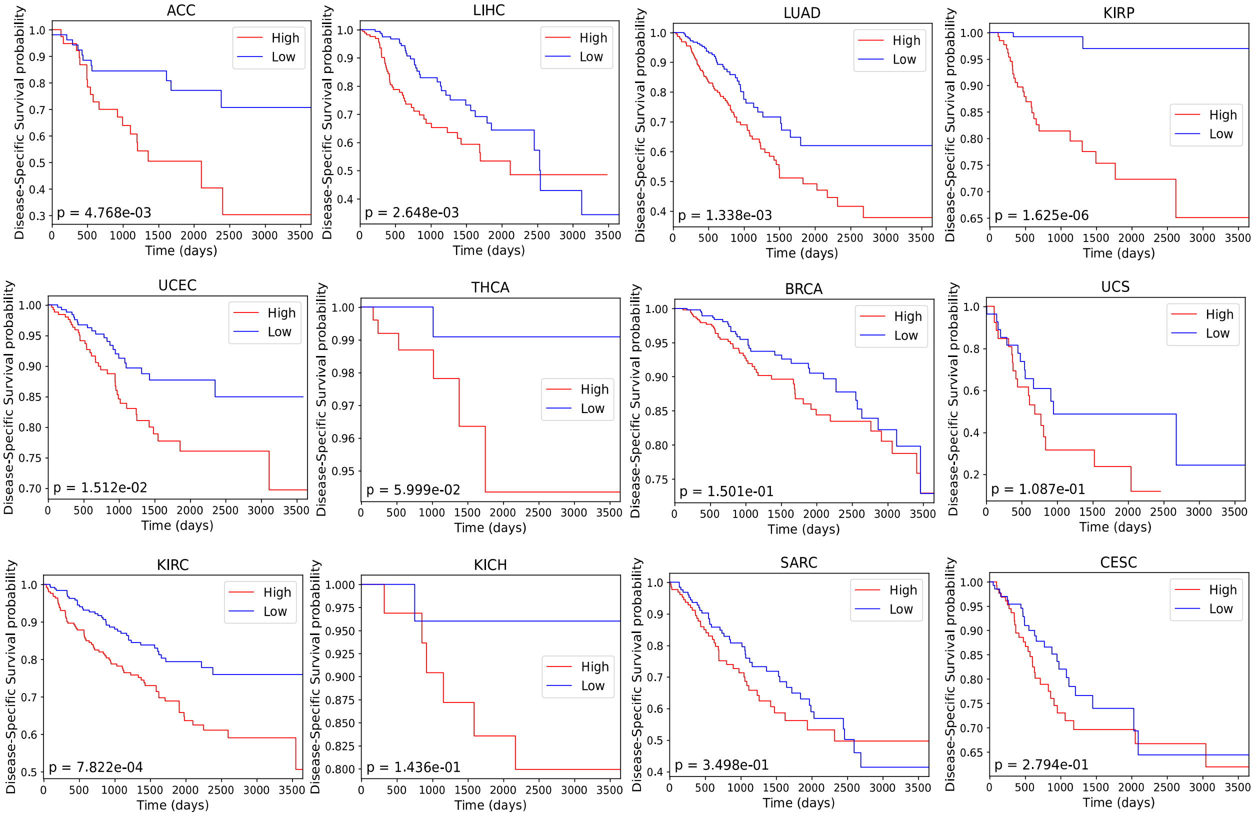

Supplementary Figure 2. Disease-specific survival across TCGA tumor types stratified by PanRibo-515 score.

Each panel is labeled with the corresponding tumor type and compares "High" (red) versus "Low" (blue) PanRibo-515 score groups defined by the median PanRibo-515 value per cancer. Disease-specific survival was evaluated using the Python lifelines package (alongside pandas, numpy, and matplotlib), and p-values in each subplot were derived from log-rank tests.

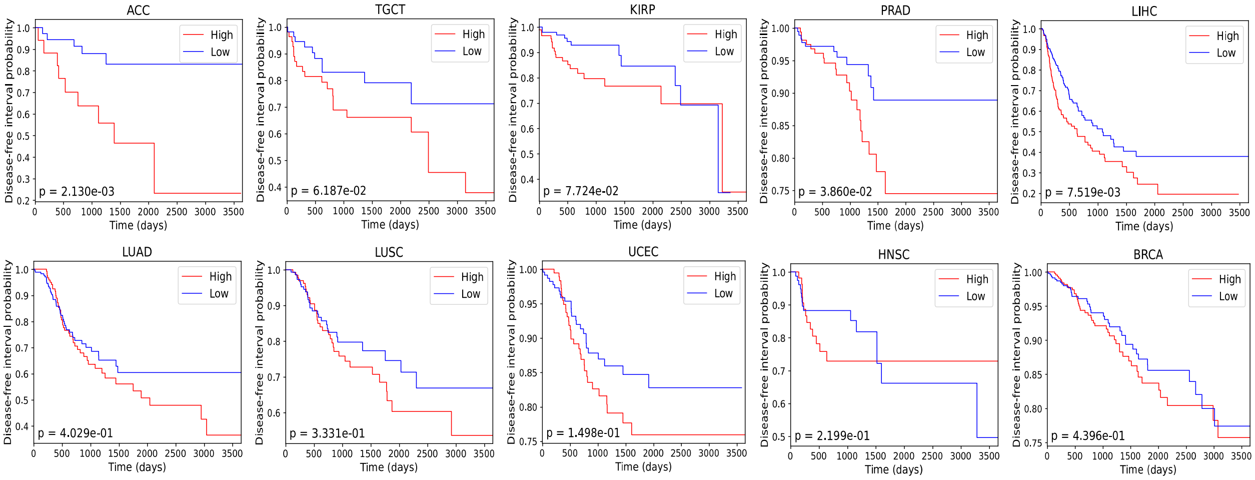

Supplementary Figure 3. Disease-free interval across TCGA tumor types stratified by PanRibo-515 score.

Each panel is labeled with the respective tumor type. Patients were divided into “High” (red) and “Low” (blue) PanRibo-515 score groups according to the median PanRibo-515 value per cancer. Disease-free interval curves were generated using Python’s lifelines package (together with pandas, numpy, and matplotlib), with log-rank tests used to determine the p-values shown in each plot. These data emphasize the potential influence of RiBi activity on disease-free outcomes across multiple tumor types.

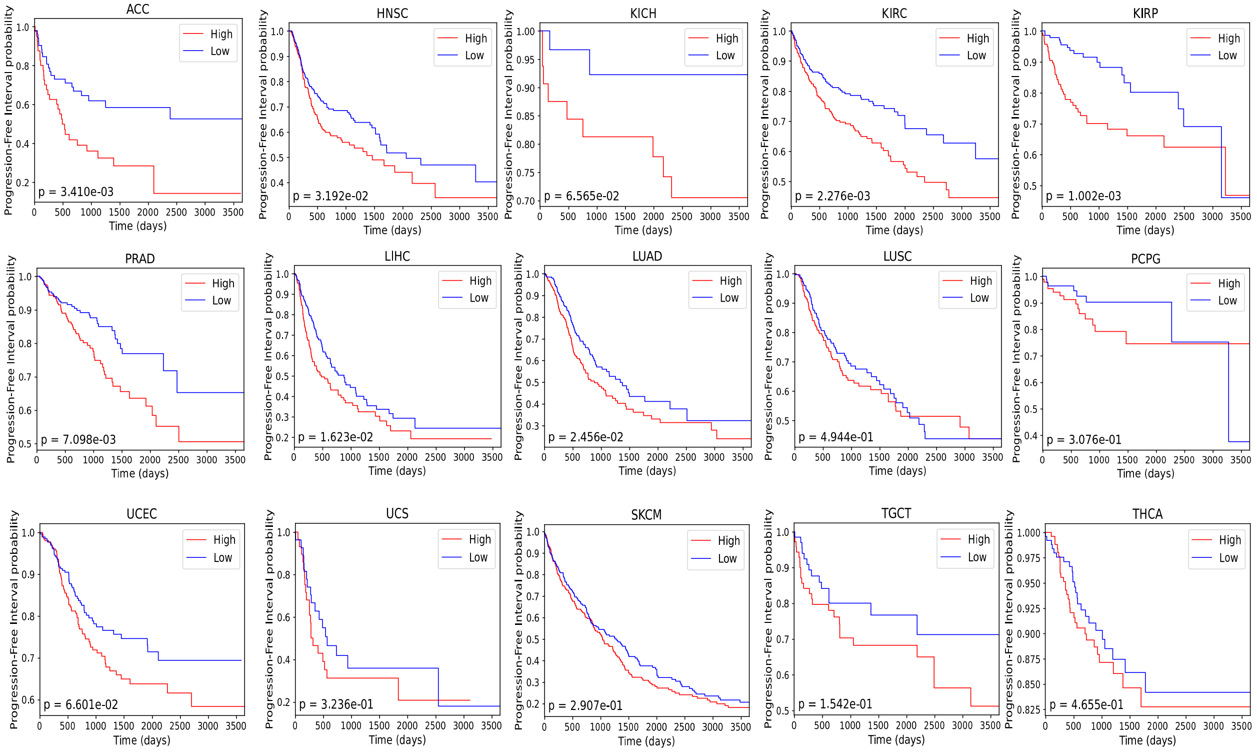

Supplementary Figure 4. Progression-free interval across TCGA tumor types stratified by PanRibo-515 score

Each panel is labeled with the specific tumor type, distinguishing “High” (red) versus “Low” (blue) PanRibo-515 score groups according to the median PanRibo-515 score per cancer. Curves were generated using Python’s lifelines package (alongside pandas, numpy, and matplotlib), and p-values were obtained from log-rank tests. These data suggest that heightened RiBi activity is associated with progression-free outcomes across several malignancies.

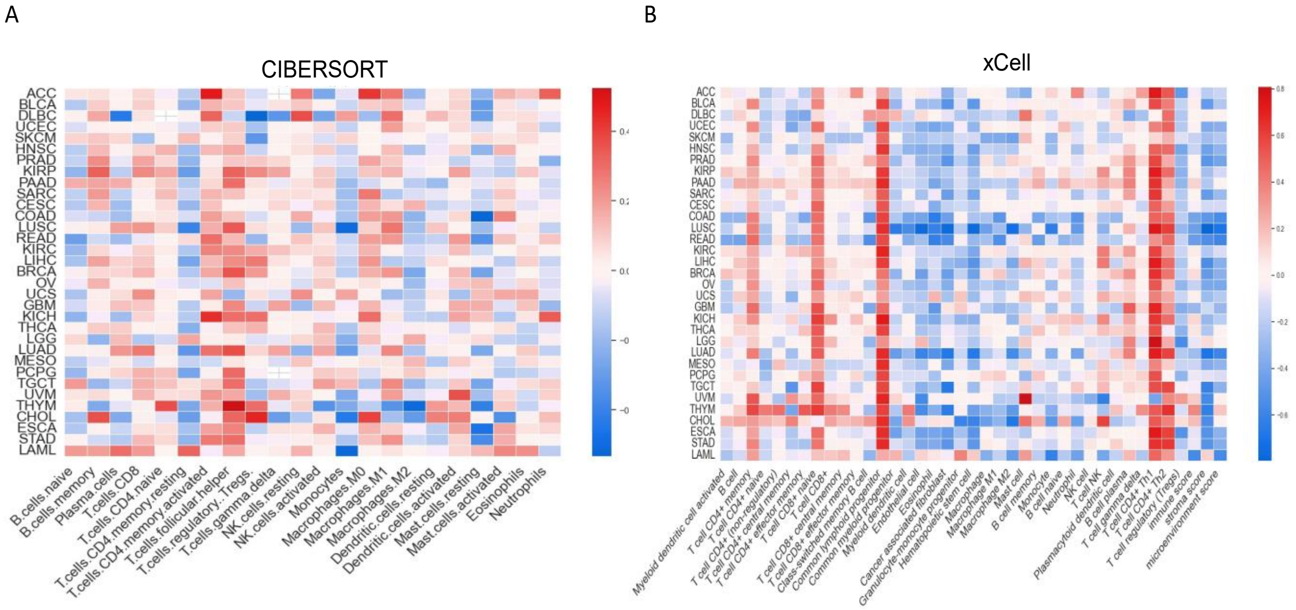

Supplementary Figure 5. Correlation of PanRibo-515 Score with Immune Cell Populations across Cancer Types.

Heatmaps illustrate Pearson's correlation coefficients between the PanRibo-515 score and various immune cell populations, calculated using immune deconvolution algorithms. (A) CIBERSORT and (B) xCell methods were applied to bulk tumor RNA-seq data across multiple cancer types from TCGA cohorts. Cancer types are indicated on the y-axis, while immune cell subsets are listed on the x-axis. Red indicates positive correlations, and blue indicates negative correlations, with color intensity indicative of the correlation strength.

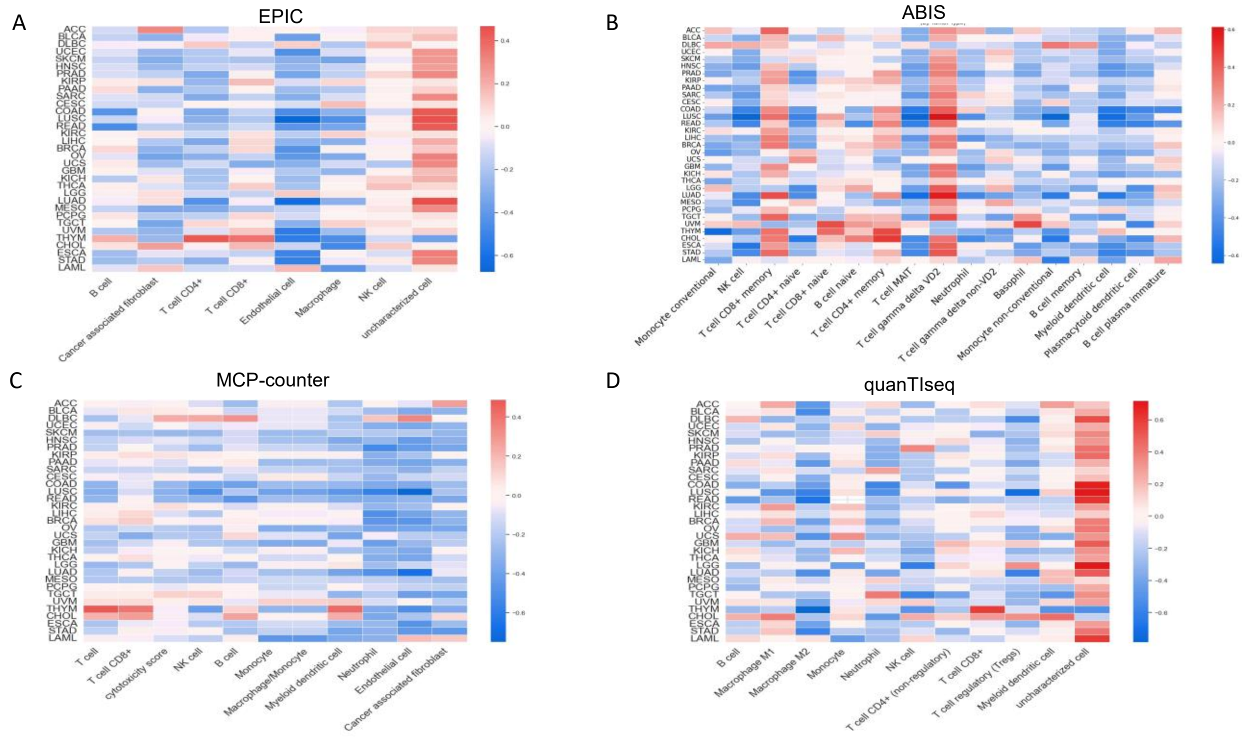

Supplementary Figure 6. PanRibo-515 Score Associations with Immune Cell Subtypes across Cancer Types.

Heatmaps depict Pearson's correlation coefficients between the PanRibo-515 score and immune cell populations as determined by multiple immune deconvolution algorithms: (A) EPIC, (B) ABIS, (C) MCP-counter, and (D) quanTIseq. Rows represent different cancer types from the TCGA dataset, and columns indicate immune cell subsets. The color scale ranges from red (positive correlation) to blue (negative correlation), with color intensity corresponding to correlation strength.

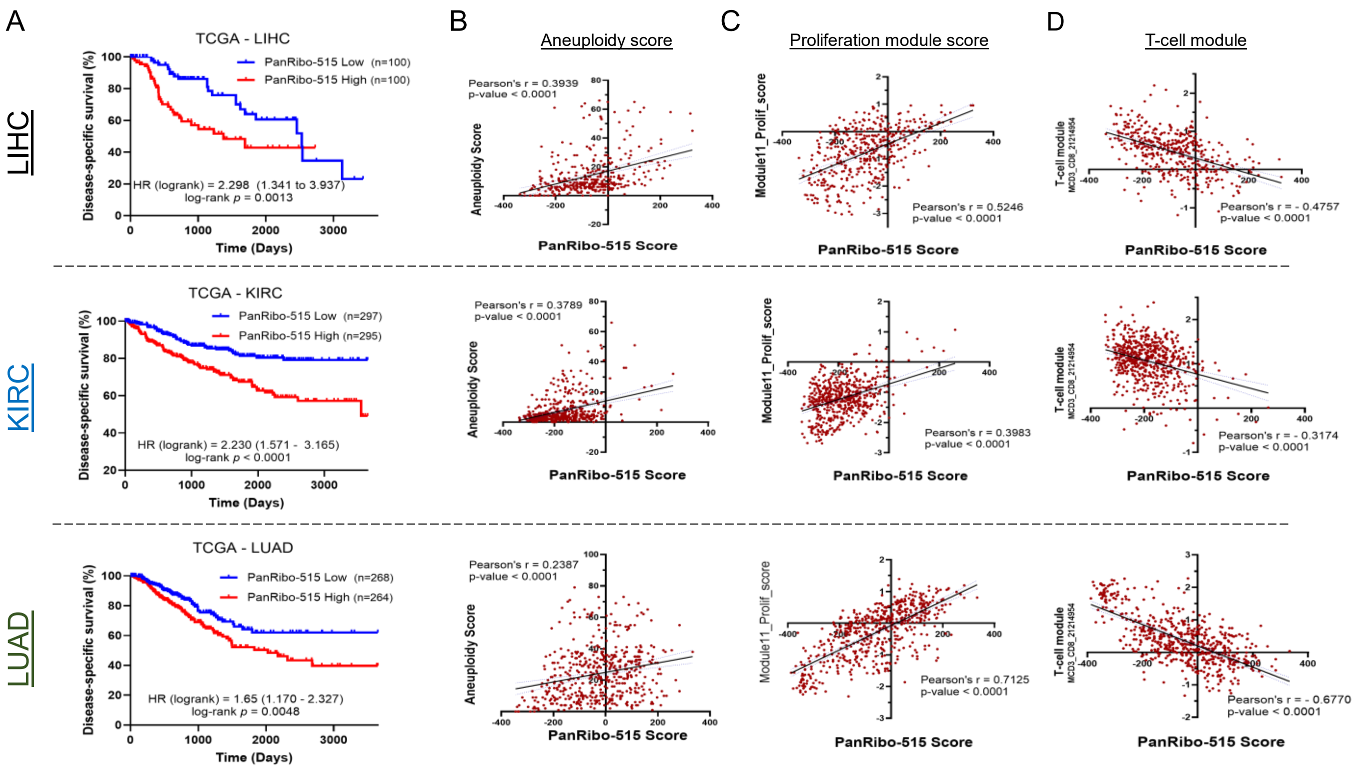

Supplementary Figure 7. PanRibo-515 score correlates with aneuploidy, proliferation, and immune cell profiles in LIHC, KIRC, and LUAD.

(A) Kaplan–Meier analysis illustrating disease-specific survival outcomes for hepatocellular carcinoma (LIHC), kidney renal clear cell carcinoma (KIRC), and lung adenocarcinoma (LUAD) patients from the TCGA cohort. Patients were categorized into "High" (red curves) and "Low" (blue curves) groups based on median PanRibo-515 scores. Elevated PanRibo-515 scores significantly correlated with reduced disease-specific survival, as indicated by hazard ratios (HR) and log-rank test p-values.

(B–D) Scatter plots illustrating the relationship between PanRibo-515 scores and three tumor-related parameters in each cancer type (rows): aneuploidy score, proliferation module score, and T-cell module score. Pearson's correlation coefficients (r) and p-values are given in each panel, indicating significant positive or negative associations between RiBi activity and these genomic or immunological features.

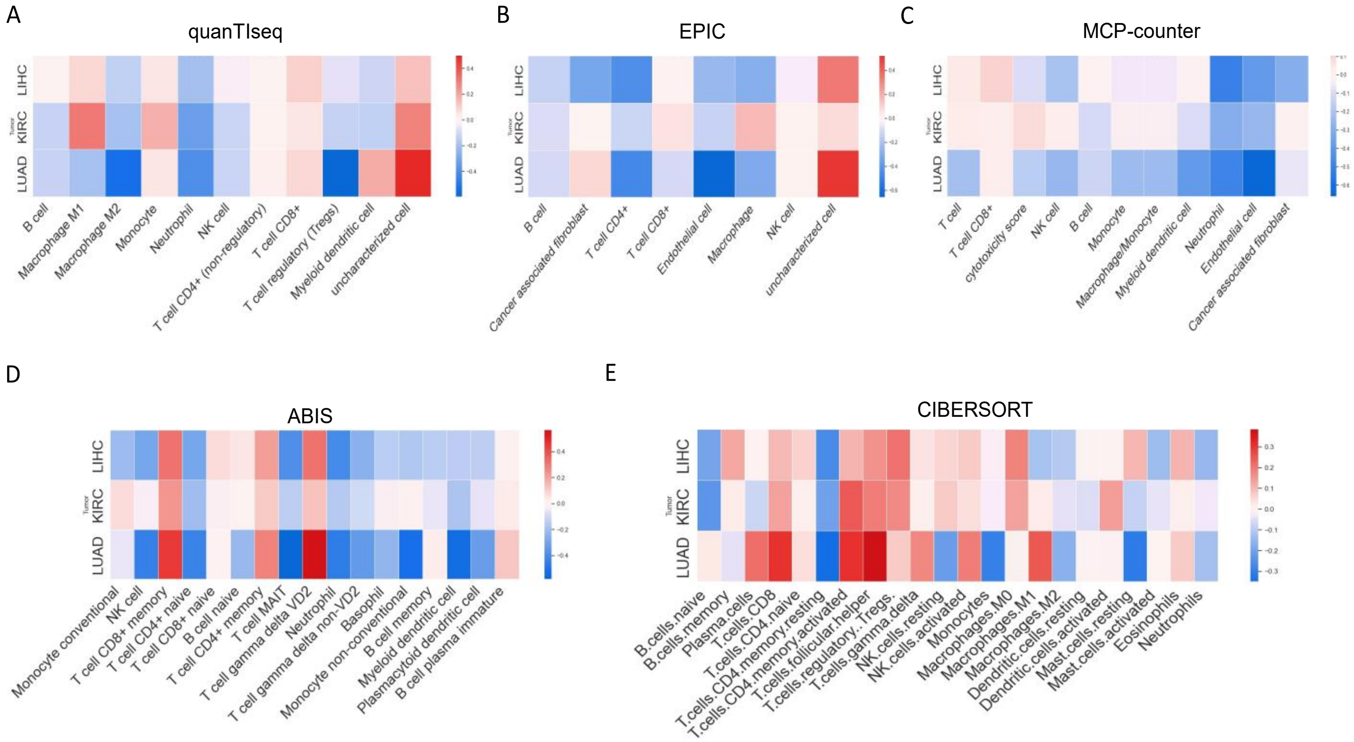

Supplementary Figure 8. Correlation of PanRibo-515 Score with Immune Cell Populations in LIHC, KIRC, and LUAD.

Heatmaps depicting Pearson correlation coefficients between PanRibo-515 scores and infiltration levels of various immune cell populations, estimated by multiple deconvolution algorithms: (A) quanTIseq, (B) EPIC, (C) MCP-counter, (D) ABIS, and (E) CIBERSORT. Positive correlations (red shades) indicate higher infiltration of immune populations associated with increased PanRibo-515 scores, whereas negative correlations (blue shades) denote reduced infiltration. These correlations vary across liver hepatocellular carcinoma (LIHC), kidney renal clear cell carcinoma (KIRC), and lung adenocarcinoma (LUAD), highlighting cancer-specific immune landscapes linked to ribosome biogenesis.

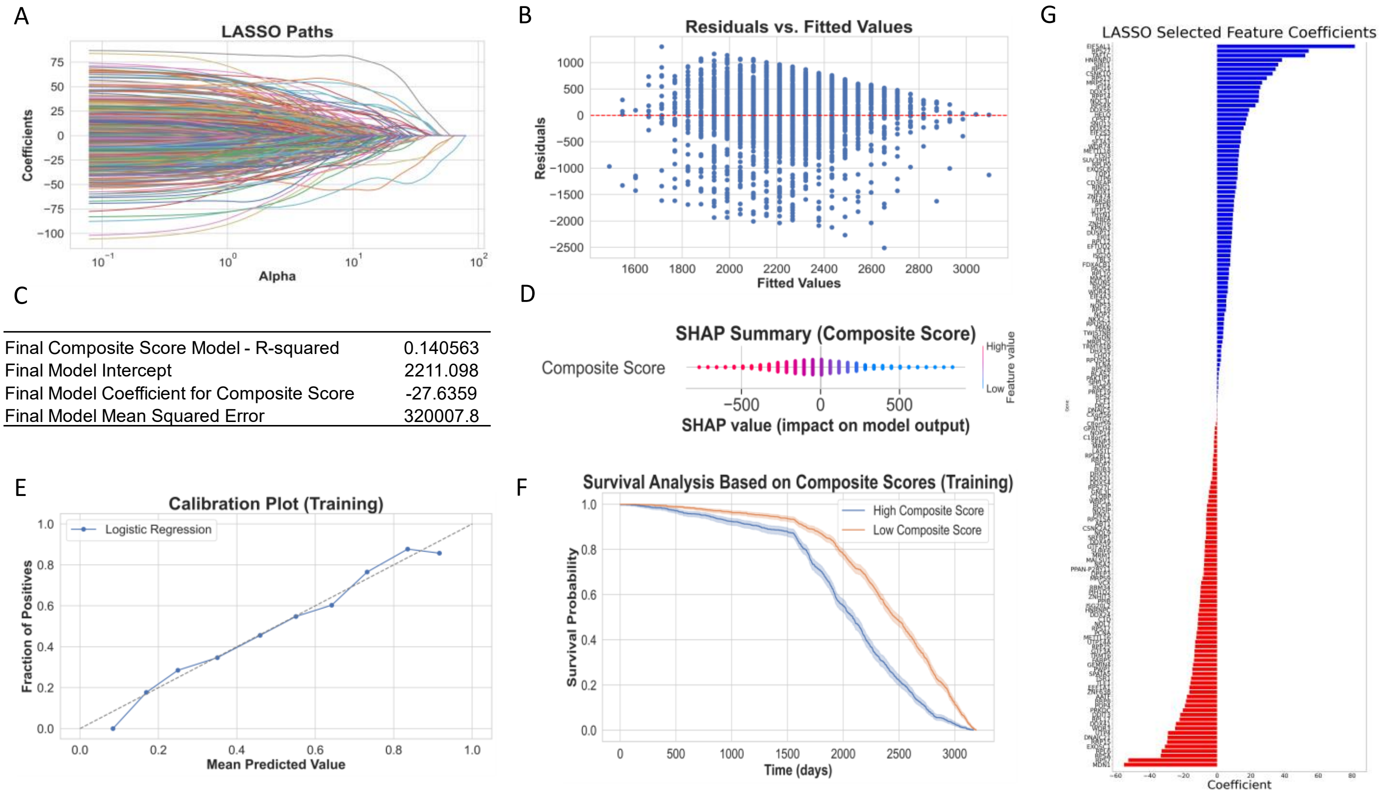

Supplementary Figure 9. LASSO-based model development and assessment using GSE202203 training data.

(A) LASSO coefficient paths plotted against varying values of the regularization parameter (α), illustrating the shrinking of coefficients as α increases.
(B) Residuals versus fitted values for the final composite score model, showing the distribution and magnitude of prediction errors.
(C) Summary statistics for the final composite score model, including R-squared, intercept, slope, and mean squared error (MSE).
(D) SHAP value distribution for the composite score, indicating how high or low score values contribute to the model's overall output.
(E) Calibration plot of the logistic regression classifier on the training set, comparing predicted probabilities to observed outcomes across deciles.
(F) Kaplan–Meier survival curves for patients stratified by high (blue) versus low (orange) composite OncoRibo-68 score, demonstrating distinct survival trajectories in the training cohort.
(G) Horizontal bar chart of nonzero LASSO coefficients, sorted by magnitude, with negative coefficients in red and positive coefficients in blue, identifying genes whose expression strongly influences survival predictions.

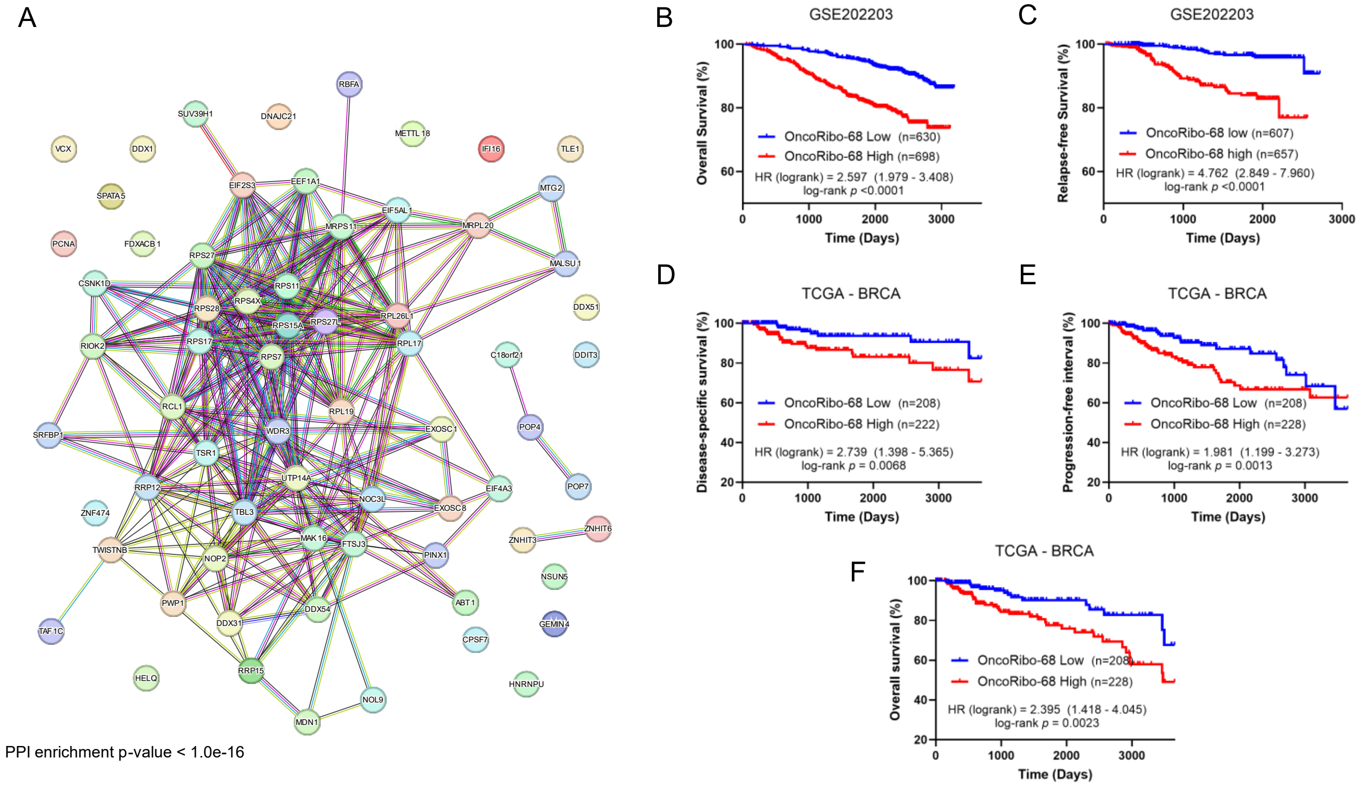

Supplementary Figure 10. Protein–protein interaction (PPI) network of OncoRibo-68 gene set and quartile-based survival analyses in GSE202203 and TCGA BRCA.

(A) PPI network of the 68 ribosome biogenesis–associated genes comprising the OncoRibo-68 score, generated using STRING with a PPI enrichment $p < 1.0e–16$. Nodes represent individual genes, and edges indicate experimentally validated or predicted interactions, forming a dense cluster of OncoRibo-68 genes.
(B, C) Kaplan–Meier curves for overall survival (B) and relapse-free survival (C) in the GSE202203 cohort, comparing patients in the top quartile (red) vs. bottom quartile (blue) of the OncoRibo-68 score. Hazard ratios (HRs) and log-rank *p*-values demonstrate significantly worse survival outcomes for those with higher OncoRibo-68 scores.
(D–F) Similar quartile-based analyses in TCGA breast cancer (BRCA) data, showing that high OncoRibo-68 score patients (red) also have poorer disease-specific survival (D), progression-free interval (E), and overall survival (F) compared to the low-score group (blue). All survival comparisons were performed using log-rank tests, and the final cohorts for each comparison are noted on the plots.

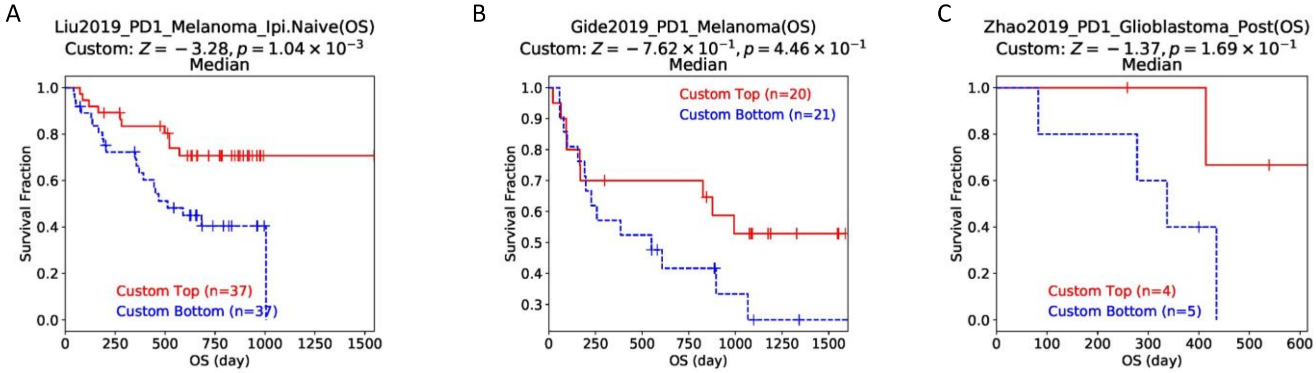

Supplementary Figure 11. Kaplan–Meier plots from TIDE analyses of multiple immunotherapy cohorts, stratified by the OncoRibo-68 gene set as a custom biomarker.

(A–C) Overall survival curves for patients receiving anti–PD-1 or anti–PD-L1 treatments in the Liu 2019, Gide 2019, and Zhao 2019 datasets, respectively. Each plot was generated using the TIDE platform (http://tide.dfci.harvard.edu/), in which "Custom Top" versus "Custom Bottom" refers to the median-based split of the OncoRibo-68 geneset's expression. Z-statistics and *p*-values are shown for each dataset, highlighting how high versus low OncoRibo-68 gene expression may impact survival in these immunotherapy-treated populations.
